# Effect of microencapsulation on *Saccharomyces cerevisiae var. boulardii* viability in the gastrointestinal tract and level of some blood biochemical factors in wistar rats

**Published:** 2019-04

**Authors:** Hassan Ghorbani-Choboghlo, Donya Nikaein, Ali-Reza Khosravi, Reza Rahmani, Zohreh Farahnejad

**Affiliations:** 1 Department of Medical Mycology, AJA University of Medical Sciences, Tehran, Iran; 2 Mycology Research Center, Faculty of Veterinary Medicine, University of Tehran, Tehran, Iran; 3 Department of Microbiology and Immunology, Faculty of Veterinary Medicine, University of Tehran, Tehran, Iran; 4 Department of Biochemistry, School of Medicine, Tehran University of Medical Sciences, Tehran, Iran

**Keywords:** *Saccharomyces cerevisiae var. boulardii*, Probiotic, Microencapsulation, Blood biochemical factors

## Abstract

**Background and Objectives:**

Probiotics are live microorganisms that, when administered in an adequate amount, confer a health benefit on the host through the gut. *Saccharomyces cerevisiae* is a widespread yeast found in nature. This microorganism has been used as a probiotic agent in recent years. In this study, the effect of microencapsulation on survival rate of *S. cerevisiae var. boulardii* in the simulated gastrointestinal tract medium and the impact of microencapsulated *S. cerevisiae var. boulardii* on some serum biochemical factors in a rat model was evaluated.

**Materials and Methods:**

30 male wistar rats were divided into three groups (control, rats receiving microencapsulated *S. cerevisiae var. boulardii*, and rats receiving *S. cerevisiae var. boulardii* alone). The probiotic was gavaged at a dosage of 2 gr/kg BW for 8 weeks. Blood was collected from rats at the end of the treatment period and biochemical factors were measured using Mancompany kits.

**Results:**

The results showed a significant increase in viability of microencapsulated *S. cerevisiae var. boulardii* in comparison with free *S. cerevisiae var. boulardii* (p<0.05). Weight of rats in probiotic treated groups was significantly higher in comparison with the control group (p<0.05). Moreover, probiotic treatment reduced mean levels of triglycerides, cholesterol, free blood sugar and liver enzymes in rats.

**Conclusion:**

Microencapsulation could increase the survival rate of yeast probiotics in the gastrointestinal tract; however, more studies are needed for better understanding of the exact effect of microencapsulation on probiotics’ function.

## INTRODUCTION

Probiotics, as defined by WHO and FAO, are “live microorganisms which when administered in adequate amounts confer a health benefit on the host” (1). According to literature, the main benefit of probiotics is maintaining a healthy digestive tract and immune system (2–4). Today, there are many commercially available probiotics developed from different species of nonpathogenic bacteria specially *Lactobacillus* spp. and *Bifidobacterium* spp. There are also some nonbacterial probiotics including some yeast species (5).

The genus *Saccharomyces* is widespread yeast found in nature. This microorganism has been used as a probiotic agent in recent years (6). *Saccharomyces boulardii*, found in probiotic preparations, is now a strain of *S. cerevisiae* known as *S. cerevisiae var. boulardii* (7). Recent studies have shown that these two species are genetically identical (8). *S. boulardii* is known for its anti-inflammatory, immunomodulatory and microbiome regulation effects and it has been approved by food and drug administration (FDA) as generally regarded as safe (GRAS) (1). It has been shown that this yeast inhibits microbial translocation and secretion of inflammatory cytokines such as IL6 (2). This yeast can survive inside the gastrointestinal tract because it can endure high temperatures and low pH (3). It is used for treatment of diarrhea as well (4).

Although many studies focus on improving advantages of probiotics, it must be noted that the most important characteristic in a probiotic microorganism is to survive stomach passage and preserve its function and viability in the intestinal tract (5, 6). Thus, methods improving survival rate of probiotics have high priority. For this purpose, this study was designed to investigate the effect of microencapsulation on survival rate of *S. boulardi* in simulated gastrointestinal tract condition and the impact of microencapsulated *S. boulardi* on some blood biochemical factors in the mentioned model (7, 8).

## MATERIALS AND METHODS

### Animal model.

30 male wistar rats weighing between 100–110 gr were used in this study. Rats were prepared from the Department of Laboratory Animals, Faculty of Veterinary Medicine, University of Tehran, Tehran, Iran. Rats were kept at intermittent lightening conditions at 20°C. Standard pellet food and water was provided ad libitum. Rats divided into three groups each containing 10 rats: rats receiving microencapsulated *S. boulardi*, control group and rats receiving uncapsulated *S. boulardii*. Probiotic was gavaged to rats at the dosage of 2 gr/kg body weight for 8 weeks and Control group received normal saline (8).

### Yeast strain.

*S. boulardii* (ATCC 74068) was obtained from Mycology Research Center, Faculty of Veterinary Medicine, University of Tehran, Tehran, Iran. It was cultured on sabouraud's dextrose broth (Merck, Germany) supplemented with chloramphenicol (0.05%) (SC) and shaker incubated at 28°C, 180 rpm for 48 hrs. After incubation, cells were centrifuged at 800 × g for 10 min and washed three times with sterile phosphate buffered saline (PBS) before use.

### Microencapsulation.

Extrusion technique was used for microencapsulation of *S. boulardii* as described previously (9). In brief, cells were suspended in 2% sodium alginate solution. Then, this mixture was injected to 0.1 M calcium chloride solution using a sterile insulin syringe. Formed droplets were isolated from suspension with centrifugation.

### Viability of microencapsulated/ un-encapsulated *S. boulardii*.

A gastrointestinal simulation environment (6) was used to evaluate the viability of microencapsulated/un-encapsulated *S. boulardii*. Cells were incubated in simulated medium with pH adjusted to 2 and 8 for 120 min at 28°C. Samples were collected from suspension at 0, 30, 60 and 120 min after incubation. Total count was done by preparing 10 fold serial dilutions in peptone water culturing samples on SC agar at 28°C for 72 hrs. Viability was determined by determining the number of yeast cells grown on SC to Control (time 0).

### Determining blood biochemical factors.

Blood was collected from rats at days 0 and after probiotic treatment. Biochemical factors including free blood sugar, cholesterol, triglycerides, and liver enzymes (SGOT and SGPT) were measured using Man company (name of company, city, country??) kits according to the manufacturer's instructions and autoanalyser.

### Statistical analysis.

Data were analyzed using SPSS version 21. Analysis of variance (ANOVA) was used to compare the results and p value of less than 0.05 considered significant.

## RESULTS

### Size and shape of beads.

**Fig. 1.**
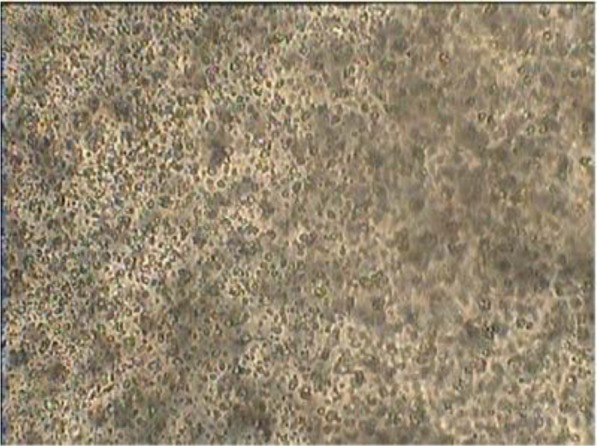
Cross-section and internal appearance of yeast coated beads (×400 magnification)

Surface morphology and shape of the 50 randomly selected calcium alginate beads were determined using light microscopy at magnification of 400×. The shape of the beads was generally spherical, sometimes elliptical with a mean diameter of 50–90 μm. Internal appearance of coated beads at 400× showed that yeasts were distributed randomly in the alginate matrix (Fig. 1). The loss during encapsulation was very low due to gentle methods used.

### Cell tolerance to gastrointestinal conditions and determination of total viable counts.

Survival rate of encapsulated yeast cells in simulated gastric pH and intestinal bile salt solutions is shown in Table 1. No significant reduction in viable count was observed in free as well as encapsulated cells in distilled water (pH 6.5) after incubation for 2 hrs (control) (p>0.05). However variations in counts of free (un-encapsulated) and microencapsulated *S. cerevisiae* var *boulardii* during incubation in simulated gastric juices (SGJ) and simulated intestinal juices (SIJ) were significant (p<0.05). Although, results demonstrated that there were significant reductions in the number of free yeasts after immediate exposure to pHs 2 and 8, during 120 min incubation in simulated gastric and intestinal condition there was a significant increase in survival rate of yeast cells in the microcapsules compared to free cells (p < 0.05).

**Table 1.** Viability of microencapsulated and unencapsulated *S. boulardii* in semi gastrointestinal condition in different times.

**Media condition**	**Time (min)**	**0**	**30**	**60**	**90**	**120**	**Reduction amount**
Acidic	uncapsulated	10	8	6	5	2.4	7.6
	microencapsulated	10	10	8	7.3	6.1	3.9
Alkaline	uncapsulated	10	7	5	4	2	8
	microencapsulated	10	9	7	6	6.4	3.6

Viability of *S. boulardii* was determined in semi gastrointestinal conditions in acidic and basic pH in different times. The results showed a significant increase in viability of microencapsulated *S. boulardii* in comparison with un-encapsulated *S. boulardii* (p<0.05) (Table 1). Reduction of *S. boulardii* count in basic media was lower than acidic condition. However this reduction was not significant (p>0.05).

### Animals’ weight gain.

Rats were weighted at the end of experiment. Weight of rats in probiotic treated groups was significantly higher in comparison with control group (p<0.05) (Table 2). Liver weight was more in probiotic treated groups however this weight gain was not statistically significant (p>0.05).

**Table 2.** Mean total weight and liver weight of rats studied after 8 weeks of treatment with microencapsulated/ un encapsulated probiotic

**Group**	**Mean weight (gr)**	**Liver weight (mean)**
Control	146.8	2.7
Unencapsulated treated	176	3.4
Microencapsulated treated	184.6	3.9

### Measurement of blood biochemical factors.

Table 3 demonstrates the mean amount of certain biochemical factors in blood of different rats. Probiotic treatment reduced mean level of TGs, cholesterol, free blood sugar and liver enzymes in studied rats. These reductions were not significant between microencapsulated/un-encapsulated probiotic treated rats (p>0.05) whilst in comparison with control group significant reduction was seen (p<0.05).

**Table 3.** mean of studied biochemical factors’ level in blood of different groups

**Biochemical factor**	**Control**	**Microencapsulated**	**Un encapsulated**
Trigelycerides (mg/dl)	76.3	62.3	58.2
Cholesterol (mg/dl)	62.7	57.3	51.2
Free Blood Sugar (mg/dl)	146	132	118
SGPT/ALT (U/L)	94	82	78
SGOT/AST (U/L)	162	151	143.2

## DISCUSSION

*Saccharomyces boulardii* has been known as a nonpathogenic yeast with biotherapeutic effects (17). The number of viable yeasts in the intestinal tract is critical to achieve yeasts efficacy, yet many of yeast cells do not survive the gastrointestinal tract (18). To maintain viability and function of probiotic, microencapsulation would be an appropriate method.

In the present study, effect of alkaline and acidic environment on viability of microencapsulated *S. boulardii* was investigated. According to the literature, these conditions are important in metabolic functions of microorganisms (19). In our study, alkaline pH (8) showed better results than acidic pH. However this was not significant in microencapsulated *S. boulardii*. Duongthingoc et al. (2013) revealed that alkaline pH would restrict rapid proliferation of *S. boulardii* but maintain its CFU count (19). We demonstrated a reduction in all experiments 120 minutes after staying in an acidic and alkaline condition. The greatest reduction amount was for un-encapsulated *S. boulardii* in alkaline media, which shows lack of proliferation in this group. However, microencapsulation could improve yeast survival rate in different pH. Arslan et al. (2015) investigated the efficacy of different materials including gelatin, whey protein concentrate, modified starch, maltodextrin, pea protein isolate and gum arabic for microencapsulation of *S. boulardii*. Gum Arabic followed by gelatin and pea protein had the best survival rates at different pH levels (10). In our study, microencapsulation increased survival rate of yeast probiotic, however with increasing the exposure time, the survival rate decreased; which is compatible with other studies (10, 20).

Rats in the probiotic treated group had significantly more weight gain than the non-treated. Probiotics inhibit overgrowth of pathogens, modify gut microbiota and increase pancreatic digestive enzymes. These functions affect absorption of nutritional substances from intestines (21–23). More weight gain in *S. boulardii* treated rat would be as a result of this phenomenon. The role of yeast probiotics in protecting the liver is well known (24). *S. cerevisiae* var *boulardii* could improve liver function in rats treated by dimethyl nitrosamine (DMN) significantly (24). In another study, a fermented substance from *S. cerevisiae* could protect rat liver from carbon tetrachloride (CCL_4_) (25). In the current study, liver weight none significantly increased in probiotic treated rats in comparison with control groups and the amount of liver enzymes had decreased in probiotic treated rats. This is in concordance with other studies. We did not find significant differences in liver function between microencapsulated/un-encapsulated *S. boulardii*. Studies have shown that probiotic consumption would control hyperglycemia (26). This is similar to our findings in which rats fed with *S. boulardii* diet had lower levels of FBS. In addition, the amount of triglycerides and cholesterol in plasma had decreased as well which has been shown by other studies too (27, 28).

## CONCLUSION

In conclusion, microencapsulation could increase survival rate of *S. boulardii* at different pH levels. Although microencapsulation could improve the effect of yeast probiotics on blood biochemical factors, we couldn't find significant differences in tested factors. Thus, more studies are needed to evaluate the effect of microencapsulation on *S. boulardii* functions *in vivo*.
